# Enhanced Production of (S)-2-arylpropionic Acids by Protein Engineering and Whole-Cell Catalysis

**DOI:** 10.3389/fbioe.2021.697677

**Published:** 2021-07-07

**Authors:** Xiaolong Liu, Meng Zhao, Xinjiong Fan, Yao Fu

**Affiliations:** ^1^Hefei National Laboratory for Physical Sciences at the Microscale, CAS Key Laboratory of Urban Pollutant Conversion, Anhui Province Key Laboratory of Biomass Clean Energy, iChEM, University of Science and Technology of China, Hefei, China; ^2^School of Basic Medical Sciences, Anhui Medical University, Hefei, China

**Keywords:** bHSL family esterase, NSAIDs, enantioselectivity, rational design, whole-cell catalysis

## Abstract

Esterases are important biocatalysts for chemical synthesis. Several bHSL family esterases have been used to prepare (*S*)-2-arylpropionic acids with stronger anti-inflammatory effects *via* kinetic resolution. Here, we presented the discovery of key residues that controlled the enantioselectivity of bHSL family esterases to ethyl 2-arylpropionates, through careful analysis of the structural information and molecular docking. A new bHSL family esterase, Est924, was identified as a promising catalyst for kinetic resolution of racemic ethyl 2-arylpropionates with slight (*R*)-stereopreference. Using Est924 as the starting enzyme, protein engineering was conducted at hotspots, and the substitution of A203 was proved to enhance the enantioselectivity. The stereopreference of the mutant M1 (A203W) was inverted to ethyl (*S*)-2-arylpropionates, and this stereopreference was further improved in variant M3 (I202F/A203W/G208F). In addition, the optimal variant, M3, was also suitable for the resolution of ibuprofen ethyl ester and ketoprofen ethyl ester, and their efficient (*S*)-isomers were synthesized. Next, the whole-cell catalyst harboring M3 was used to prepare (*S*)-ketoprofen. (*S*)-ketoprofen with 86%ee was produced by whole-cell catalyst with a single freeze-thaw cycle, and the cells could be reused for at least five cycles. Our results suggested that Est924 variants could kinetically resolve economically important racemates for industrial production and further offer the opportunity for the rational design of enzyme enantioselectivity. Moreover, it is an economical process to prepare optically pure (*S*)-ketoprofen and (*S*)-naproxen by using an engineered strain harboring M3 as the catalyst.

## Background

Chirality is one of the essential properties of natural substances, and enantiomers are ubiquitous in nature. Different enantiomers of a biologically active molecule generally have different physiological properties, such as different degrees or different types of biological activities, or even opposite bioactivities or toxic side effects (Lough et al., 2012). Non-steroidal anti-inflammatory drugs (NSAIDs) are the world’s most used drugs with anti-inflammatory, analgesic, and antipyretic effects. 2-arylpropionate drugs (known as profen drugs) are a very important class of chiral drugs in NSAIDs, including ibuprofen, naproxen, ketoprofen, flurbiprofen, fenoprofen, and loxoprofen, among others. Profen drugs contain a stereogenic center at the carbon alpha of the carboxyl function and therefore racemic (*R*)- and (*S*)-enantiomers which are equivalent in mass. A large number of pharmacological studies on the relative activities of the two enantiomers showed that (*S*)-enantiomers have significantly higher clinical effects, and dexketoprofen, (*S*)-ibuprofen and (*S*)-naproxen are most widely used commercially (Toledo and Briand, 2018).

Due to differences in physiological functions of isomers, the Food and Drug Administration (FDA) requested manufacturers to separate each enantiomer for specific usage (Tomaszewski and Rumore, 1994). Biocatalysts have been under development for several decades to replace chemical-based synthesis because they have advantages in economic feasibility and environmental friendliness (Sheldon and Woodley, 2018). Carboxylesterases (E.C. 3.1.1.1), as the main group of natural biocatalysts for the cleavage and formation of the ester bond, has good stability, wide substrate specificity, and high chemoselectivity, stereoselectivity, and regioselectivity. It is widely used as an environmentally friendly biocatalyst for the synthesis of active pharmaceutical intermediates and fine chemicals (Romano et al., 2015). Several research groups have already described enzymatic routes for the production of (*S*)-2-arylpropionates (Sunna et al., 2002; Kim et al., 2003; Sehgal and Kelly, 2003; Kim and Lee, 2004). Recently, Vega and coworkers screened and characterized the metagenomic-derived bHSL family esterase EstD11, which has a broad substrate scope including naproxen ester and ibuprofen ester (Miguel-Ruano et al., 2021). Directed evolution technology is commonly used to improve the activity and selectivity of enzymes, and is also suitable for enhancing the enantioselectivity of esterases in the kinetic resolution of 2-arylpropionate esters (Kim et al., 2015, 2017; Ngo et al., 2019). Esterases are thus potential biocatalysts for the synthesis of NSAIDs with high optical purity. Industrial enzymes usually need to have a broad substrate scope (Adams et al., 2019). However, each reported esterase was able to synthesize only one or two NSAIDs drugs, which has a negative impact on their application.

Inspired by the broad substrate scope of the bHSL carboxylesterases (Martínez-Martínez et al., 2018) and high pharmaceutical activity of (*S*)-2-arypropionic acids, we screened key residues that controlled the enantioselectivity of bHSL carboxylesterases to ethyl 2-arylpropionates, *via* careful analysis of the structural information and molecular docking. Subsequently, Est924 (GenBank accession number MW460906), a bHSL family esterase, was set as a starting template for enzyme engineering. It was a promiscuous esterase cloned from a metagenomic library of bamboo root-soil which can hydrolyze phthalates (Liu et al., 2020) and ethyl 2-arylpropionates with slight (*R*)-stereopreference. The hotspots were identified through rational design and mutated by site-directed mutagenesis. The enantioselectivity of mutant M1 (A203W) was inverted to the (*S*)-naproxen ethyl ester. Variant M3 (I202F/A203W/G208F) resolved racemic naproxen ethyl ester with higher enantioselectivity and produced (*S*)-naproxen with 91%*ee*_p_. Furthermore, M3 was confirmed to resolve another ethyl (*S*)-2-arylpropionates with high selectivity. The products dexketoprofen and (*S*)-ibuprofen were obtained with 95%*ee* and 74.5%*ee*, respectively. This is the first report that esterases can be used in the synthesis of several NSAIDs drugs, including (*S*)-ibuprofen, (*S*)-naproxen and dexketoprofen.

Compared to the purified enzyme and immobilized enzyme, whole-cell catalyst was more readily prepared, and cell envelopes can be beneficial to stabilize enzymes. Whole-cell catalysis is widely applied as an alternative to conventional chemical methods for pharmaceutical synthesis (Lin and Tao, 2017). Through a simple freeze-thaw process, the cells were highly active and readily usable for the synthesis of (*S*)-ketoprofen and (*S*)-naproxen. Our results provide the molecular basis of the enantioselectivity of bHSL family esterases against ethyl (*S*)-2-arylpropionates, indicating a breakthrough in the development of esterases.

## Materials and Methods

### Construction of the Designed Mutants

Selected residues of Est924 were mutated by site mutagenesis using primers in Supplementary Table 1 with wild-type Est924 plasmid as a template. The introduction of single site mutations into Est924 was constructed using the QuickChange kit (Stratagene, CA, United States) by following the manufacturer’s instructions. The PCR product was incubated with DpnI (New England Biolabs, MA, United States) at 37°C for 1 h to degrade the parental template. Variants were transformed into *Escherichia Coli* BL21 (DE3) competent cells. Since naproxen is sold as the (S)-enantiomer, the recombinants were picked up and inoculated on lysogeny broth (LB) solid mediums containing 10 mM racemic naproxen ethyl ester and then cultured at 37°C for 12 h. We picked recombinants with translucent zones and those with transparent zones smaller than Est924. The standard expression and purification conditions were followed to obtain mutant enzymes. The racemic naproxen ethyl ester was subsequently used as substrate and the enantiomeric excess (*ee*) of products was analyzed by HPLC. The extracted product was purified by flash column chromatography with petroleum ether and ethyl acetate to separate acid and ester. The acid was esterified by the previously described method (Jin et al., 2003) and the *ee* of the product was analyzed using suitable column with the chiral stationary phase. (Supplementary Table 2) mounted on HPLC (Agilent, CA, United States) and UV detector at 214 nm (Godinho et al., 2012). The enantioselectivity of the esterase was expressed as the enantiomeric ratio (*E*) and calculation of *E*-value was performed using the method of Kim et al. (2017). Finally, the genes of the enzymes with increased enantioselectivity were verified by DNA sequencing (Sangon Biotech, Shanghai, China).

### Expression and Purification of Est924 and Variants

A single colony was picked up and grown in LB supplemented with kanamycin (50 μg/mL). The overnight culture was used to inoculate main cultures (800 mL LB medium containing 50 μg/mL kanamycin) to an initial OD600 of 0.05. Cells were grown at 37°C to an OD600 of 0.6, and IPTG was added to a final concentration of 0.1 mM. The cultures were incubated at 30°C for an additional 10 h, and cells were harvested by centrifugation at 10,800 *g* at 4°C for 20 min. Cell pellets were washed with a NaCl solution (0.9%, w/v) and frozen at −20°C for later purification.

The cell pellets were thawed on ice, resuspended in 10 ml lysis buffer (50 mM phosphate buffer containing 300 mM NaCl and 10 mM imidazole, pH 8.0) per gram of cell pellet and disrupted by sonication (Sonicator QSonica Q500 Ultra Sonicator) at 0°C. Cell debris was removed by centrifugation at 18,000 *g* for 30 min at 4°C. The supernatant was filtered through a 0.25 μm PVDF filter and was loaded onto a Ni-NTA column (Thermo scientific) equilibrated with lysis buffer. The column was then washed with six column volumes of wash buffer (50 mM phosphate buffer containing 300 mM NaCl and 20 mM imidazole, pH 8.0) after loading the filtered lysate. Finally, the protein was eluted with elution buffer (50 mM phosphate buffer containing 300 mM NaCl and 250 mM imidazole), and fractions containing target enzyme (determined by SDS-PAGE) were pooled and dialyzed at 4°C against Tris–HCl buffer (20 mM, pH 7.5). Protein expression and purity were assessed by SDS-PAGE (Supplementary Figure 1).

### Enzymatic Activity Assay

Esterase activity was measured using the standard assay: 50 μg purified esterase was reacted with 10 mM racemic ethyl 2-arylpropionates in 200 mM Tris–HCl buffer (pH 8.0) with 5% DMSO for 30 min at 30°C. The reaction was terminated by adjusting the pH to 2 using 6 M HCl and extracted with ethyl acetate. The organic layers were dried over anhydrous Na_2_SO_4_ and evaporated under vacuum. Conversion ratio was measured using NMR. The *ee* of products was analyzed by HPLC as described above.

### Structure Modeling and Molecular Docking

Homology modeling of Est924, and M3 (I202F/A203W/G208F) were performed using the SWISS-MODEL^1^ online system using esterase EstD11 (PDB id: 7AT3) as a template (sequence similarity: 49%) (Waterhouse et al., 2018; Miguel-Ruano et al., 2021). The structure of SsoEst1 was simulated in the same manner using 5LK6 as the template (sequence similarity 91.78%). The final models were validated by PROCHECK^2^ and Verify 3D (Laskowski et al., 1996). Pymol was employed to visualize and analyze the 3D structure of Est924 and its mutants (DeLano, 2002).

Est924 and M3 were docked with (*R*) and (*S*)-naproxen using the Surflex-Dock module of Sybyl-X 2.0 program, respectively. All docking calculations were set to equal parameters (20 poses each), only using the pose that was highest ranked by Surflex-Dock. The conservative degree of residues was determined by the WebLogo 3.0 tool (Crooks et al., 2004).

### Bioconversion Conditions

After induction, cells were collected by centrifugation at 8,000 × *g* for 10 min, washed twice with 0.9% brine, and resuspended in the reaction mixture containing 50 mM potassium phosphate buffer (pH 8.0), 10 mM ketoprofen ethyl ester, and 5% DMSO to form cell suspension (OD600 nm = 3) with a total volume of 10 mL (in a 50 mL shake flask). The bioconversion reactions were performed at 30°C and 200 rpm. At different time points, 100 μL of sample was taken and mixed with 4 μL 6 M HCl and extracted with EtOAc. The resulting supernatant was centrifuged at 12,000 × *g* for 5 min and analyzed by HPLC as described above. For the recycling of the catalyst, the whole cell and supernatant are separated by centrifugation at the end of the batch reaction. Add a new reaction solution to the cell and restart the catalysis process.

## Results and Discussion

### Substrate Specificity and Enantioselectivity of Est924

**FIGURE 1:**
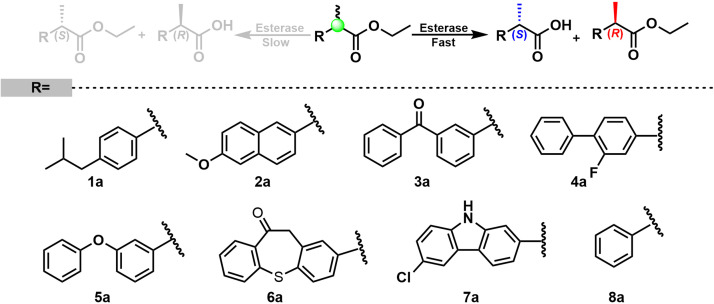
Substrate specificity and enantioselectivity of carboxylesterase Est924 for ethyl 2-arylpropionates.

It is widely known that bHSLs are promiscuous by nature (Pascale and Arnaud, 2012). For instance, esterase EH1 and EstD11 belong to the bHSL family and demonstrate promiscuous substrate specificity (Martínez-Martínez et al., 2018; Miguel-Ruano et al., 2021). Organic solvents and the alcohol moiety of esters often affect the enantioselectivity of esterases. Est25, EstD11, and Est924 have relatively low (*R*)-enantioselectivity to ethyl 2-arylpropionates, for this reason are the suitable template for the engineering of an (*S*)-enantioselective biocatalyst. Methanol, DMSO, and Triton-X100 are good cosolvents for Est924. However, the addition of methanol produces transesterified products that are difficult to be separated from the reaction mixture (data not shown). The foam formed by Triton-X100 is not conducive to industrial applications. Therefore, ethyl 2-arylpropionates and DMSO were selected as the substrates and cosolvent, respectively. The substrate specificity of Est924 was determined at 30°C in pH 8.0 buffer by using racemic ethyl (*R*, *S*)-2-arylpropionates as substrates. As shown in Figure 1 and Table 1, Est924 could hydrolyze all tested substrates, indicating that it had a wide substrate scope. In the pharmaceutical industry, there is an increasing demand for esterase-mediated chiral resolution of racemic 2-arylpropionate esters due to the high activity and low side effects of the (*S*)-enantiomers. The enantioselectivity of Est924 was subsequently determined using (*R*) and (*S*)-naproxen ethyl ester as substrates. Similar to Est25 (Kim et al., 2006) and SsoEST1 (Sehgal and Kelly, 2003), wild-type Est924 had activity toward (*R*)-naproxen ethyl ester and (*S*)-naproxen ethyl ester, with a slight preference for (*R*) -enantiomer (10%*ee*_R_).

**TABLE 1:** Hydrolytic activity of esterase Est924 for ethyl 2-arylpropionates.

Entry	Substrate	Conversion (%)^[a]^	Specific activity (mM/mg/min)	ee%^[b]^
1	1a	95	1.5	20%(*R*)
2	(*S*)−2a	90	3.3	N.D.
3	(*R*)−2a	95	3.5	N.D.
4	3a	97	3.5	5%(*R*)
5	4a	85	2.7	N.D.
6	5a	90	3.1	N.D.
7	6a	82	2.3	N.D.
8	7a	82	2.4	N.D.
9	8a	90	2.7	5%(*R*)

*^[a]^Conversions were determined by NMR.*

*^[b]^Enantiomeric excess values were determined by chiral HPLC.*

*N.D., not determined.*

### Structural Analysis of bHSL Family Esterases

**FIGURE 2:**
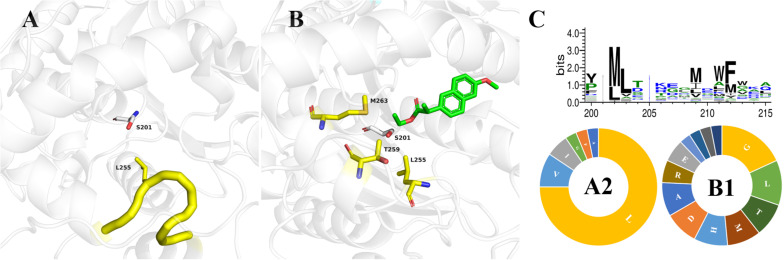
The crystal structures of Est25 and its mutant Est25_F72G/L255W_ with the docked substrate (*R*)-naproxen ethyl ester. **(A)** The catalytic serine (S201) and residue L255 of Est25 (PDB: 4J7A) were shown as white and yellow sticks, respectively. **(B)** The docked substrate (*R*)-naproxen ethyl ester was shown as green. Residues that close to the naphthalene ring were shown as blue sticks. **(C)** Conservation of residues at positions A2 and B1 in 32 crystal structures of bHSL family esterases. The distributions of 20 amino acids at positions A2 and B1 were also analyzed (below).

Enzymes with broad-spectrum substrate applicability generally owe this to their larger active pocket, similar to bHSL family esterases (Martínez-Martínez et al., 2018). According to the classification of the Esther database (Lenfant et al., 2013), 111 esterase structures of the bHSL family were resolved. We superimposed the crystal structures of Est25 (PDB: 4J7A) and 41 wild-type enzymes of bHSL family (Supplementary Figure 2). Three-quarters of bHSL family esterases had highly similar crystal structures, although the lowest sequence similarity observed between 5IQ3 and 3D7R was only 11.7% (Supplementary Table 3). The residue L255 of Est25 attracted our attention, because the variant Est25_L255W_ reversed the enantioselectivity of the racemic ketoprofen ethyl ester, resulting in (*S*)-ketoprofen with 80%*ee*_p_ (Yoon et al., 2014). Residue L255 was located at the loop between helix 10 and 11 with an isobutyl side chain extension to catalytic serine (Figure 2A). The enantiomer discrimination by enzymes is a very accurate mechanism, which often involves a few amino acids located at the active site. Based on structural information, about three-quarters of the 41 analyzed structures contain the same loop, indicating that it is conserved in the bHSL family (Supplementary Table 3). Alternatively, carboxylesterase SsoEST1 has the same residue (L198) as Est25 (L255), but selectively produces (*S*)-naproxen when racemic naproxen methyl ester was used as a substrate (Sehgal and Kelly, 2003). This indicates that other residues in the active pocket probably control the stereoselectivity of the α carbon. By docking (*R*)-naproxen ethyl ester into Est25 (Figure 2B), several residues around the Cα were selected to test whether they played a role in the stereoselectivity of the enzymes, due to the opposite stereoselectivity of Est25 and SsoEST1. As shown in Table 2, the residues at position A2 in all crystal structures were hydrophobic amino acids, and the residues at position B1 exhibited lower conservation (Figure 2C). Since residues at position A2 and position B1 directly extend their side chains toward the *α* carbon of substrates, their low conservation suggested that these residues might be responsible for the different stereoselectivity of enzymes.

**TABLE 2:** Key residues in crystal structures of bHSL family esterases.

PDB entry	A1	A2	B1	B2
Est924	I202	A203	G208	I212
SsoEST1	F197	L198	H202	F206
1EVQ	L205	L206	M210	F214
1JJI	I209	L210	I214	F218
1JKM	F259	I260	G264	L268
2C7B	S205	L206	L210	F214
2YH2	I208	L209	L213	F217
3AIK	F197	L198	H202	F206
3D7R	V202	L203	G207	I211
3DNM	L206	A207	T211	M215
3GA7	G215	L216	D220	Y224
3H17	M193	V194	G198	M202
3QH4	A209	F210	A214	M218
3WJ1	F197	L198	H202	F206
4J7A	F254	L255	T259	M263
4KRY	G215	L216	D220	Y224
4OB6	F207	L208	M212	F216
4V2I	W206	L207	A211	F215
4XVC	S194	V195	R199	I203
4YPV	F207	L208	G212	F216
5GMR	S196	V197	R201	F205
5HC0	L239	L240	N244	A248
5IQ3	F254	W255	T259	M263
5JD4	F209	L210	L214	F218
5JD5	I219	L220	D224	F228
5L2P	F203	L204	L208	Y212
5LK6	F197	L198	H202	F206
5MII	G238	L239	E243	A247
6AAE	Y202	L203	G207	F221
6I8D	F209	L210	H214	F218
6K1T	H197	L198	W201	F206
6K34	I203	L204	A208	F222
6KEU	V211	L212	S216	F220
6KMO	F228	L229	M233	F237
7AT0	M193	V194	P198	M202

### Site Analysis and Rational Design of Est924 Variants

**FIGURE 3:**
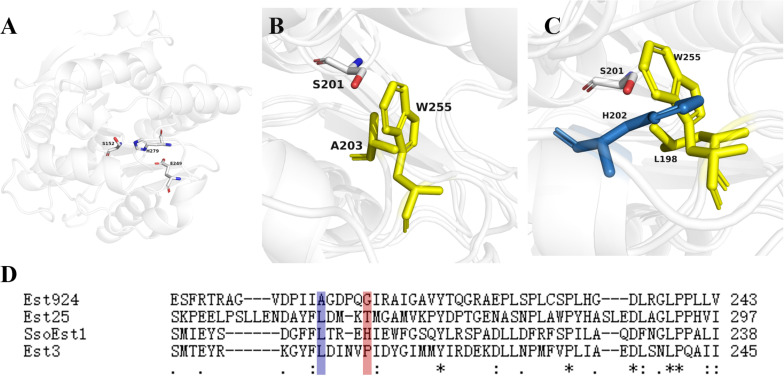
Structural analyses of modeled Est924 and reported bHSL family enzymes. **(A)** Crystal structure of modeled Est924, the catalytic residues (S152, E249, and H279) were shown as white sticks. **(B)** Superimposed the structure of Est924 and Est25_F72G/L255W_ (PDB: 5IQ3) with catalytic serine (white), residue A203 of Est924 (yellow) and W255 (cyan) of Est25_F72G/L255W_ were shown as sticks. **(C)** Superposition the crystal structures of Est25_F72G/L255W_ and SsoEst1, residues L198 (yellow) and H208 (blue) of SsoEst1, W255 (yellow) of Est25_F72G/L255W_ were shown as sticks. **(D)** Multiple sequence alignment of reported esterases for kinetic resolution of 2-arylpropionate esters. (Accession No. of Est25: Q4TZQ3, SsoEST1: A0A3G8EMI7, EST3: Q97VU2). The residues at positions A2 and B1 were indicated by blue and red boxes, respectively.

Est924 can hydrolyze a variety of ethyl 2-arylpropionates, demonstrating the same enantioselectivity as Est25. We simulated the structure of Est924 using the crystal structure of EstD11 as a template (Figure 3A). The key residues of Est924 that might control its stereopreferences were analyzed. The selectivity of Est25 mutants with L255W to (*S*)-ketoprofen ethyl ester was significantly improved, because tryptophan has the largest side chain, which increases the binding steric hindrance of the (*R*)-enantiomer (Kim et al., 2017). Superposition of the Est924 and Est25_F72G/L255W_ crystal structure (5IQ3) revealed that the A203 with a small side chain in Est924 might be responsible for the low enantioselectivity. The Est25_F72G/L255W_ residue was transferred to Est924 by A203W substitution to investigate whether the variant Est924_A203W_ could make (*S*)-naproxen as the main product (Figure 3B, yellow residues). Similarly, the structure of SsoEst1 was also simulated and superimposed with the Est25_F72G/L255W_. The imidazole group of H202 in SsoEst1 occupies the indole ring of W255, which might be the second key residue responsible for the enantioselectivity of the enzymes (Figure 3C, yellow residues). Multiple sequence alignment of reported bHSL family esterases with (R)-enantioselective resolution of 2-arylpropionates also showed that the B1 position was occupied by residues with smaller side chains (Figure 3D). Therefore, G208 at position B1 was also selected as a mutation site to prove our hypothesis. In addition, because the steric hindrance and hydrophobicity of I202 were lower than the phenylalanine in Est25 (F254), SsoEst1 (F197), and Est3 (F203), I202 was also selected for mutation. Moreover, F206 at position B2 of SsoEst1 may also be beneficial to its (*S*)-enantioselectivity, and the residue I212 of Est924 was replaced by phenylalanine.

### The Enzyme Activity and Enantioselectivity of Esterases

The enzyme activity and enantioselectivity of esterases were measured using (*R*, *S*)-naproxen ethyl ester as substrate by HPLC. Firstly, A203 was mutated to tryptophan according to the structural analysis and results obtained by Kim et al. (2017). As predicted, variation of residue A203 significantly impacts enantioselectivity, and the variant M1 (A203W) turned to (*S*)-selective, producing (*S*)-naproxen with 65.6%*ee*_p_. The variant Est924_G208F_ also reversed the stereoselectivity and hydrolyzed (*S*)-naproxen ethyl ester with moderate selectivity, yielding (*S*)-naproxen with 56.3%*ee*_p_. However, the variant Est924_I212F_ with a larger hydrophobic side chain did not affect the enantioselectivity. These results indicated that both positions (204 and 208) were effective in improving the selectivity of Est924. Variant M2 with A203W and G208F was highly (*S*)-selective, producing (*S*)-naproxen with 95%*ee*_s_. Finally, the variant with a mutation at I202 still retained the (*S*)-enantioselectivity as high as M2, and variant M3 (I202F/A203W/G208F) with 95.7%*ee*_s_ was obtained.

**FIGURE 4:**
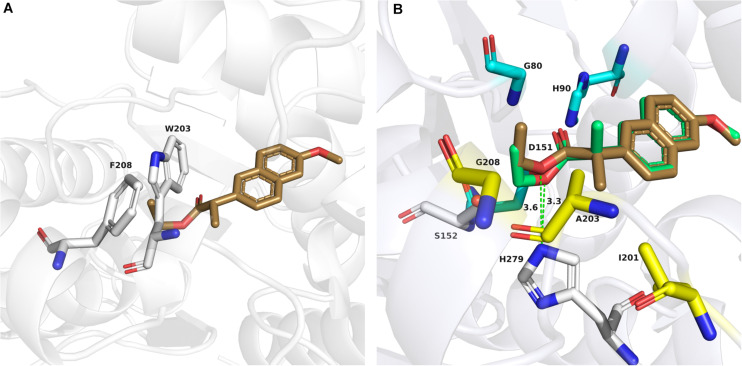
Docking models of (*R*) and (*S*)-naproxen ethyl ester to Est924 and mutant M3. **(A)** Detailed interactions of the docking (*S*)-naproxen ethyl ester (sand stick) and residues in the active site of M3 (gray stick representation). **(B)** Docking of (*R*)-naproxen ethyl ester (green stick) and (*S*)-naproxen ethyl ester (sand stick) into the active site of the Est924 model. Catalytic residues (S152 and H279) were shown as white sticks, the residues (G80, H90, and D151) forming the oxyanion hole was shown as cyan sticks. The residues around Cα of naproxen ethyl esters were shown as yellow sticks. The distance between the protonated Nε atom of the active histidine residue and the ester oxygen atom (d_NE–O_) of (*R*)- and (*S*)-naproxen ethyl ester were shown as green dashed lines.

To clarify why the enantioselectivity of the variants was reversed and increased, we investigated the specific activity (mM/mg/min) for biocatalytic reactions using wild-type Est924, M1, G208F, M2, and M3 with (*R*)-naproxen ethyl ester and (*S*)-naproxen ethyl ester as substrates. Est924 showed the lowest enantioselectivity among esterases (Table 3, list 1), which mainly caused similar hydrolysis activity as compared to the enantiomers. M1 retained 90% activity of Est924 on (*S*)-naproxen ethyl ester, and 15% activity against (*R*)-enantiomer (Table 3, list 2). Therefore, the enantioselectivity of M1 was reversed and (*S*)-naproxen was formed as the main product. M3 showed the highest enantioselectivity among the esterases examined. It was almost inactivated in its ability to process (*R*)-naproxen ethyl ester, but still retained 80% of the activity of Est924 when (*S*)-naproxen ethyl ester was used as a substrate (Table 3, list 5). Subsequently, the structure of the optimal variant M3 was simulated and docked with (*R*) and (*S*)-naproxen ethyl ester, respectively. As shown in Figure 4A, (*S*)-naproxen ethyl ester fitted well with the active pocket but failed to obtain an effective docking of (R)-enantiomer with M3. Next, we docked (*R*) and (*S*)-naproxen ethyl ester into the structure of Est924, as shown in Figure 4B. Both the (*R*) and (*S*)-enantiomer pointed their naphthalene rings to the acyl binding site region. The distance between the protonated Nε atom of the active histidine residue and the ester oxygen atom (d_NE–O_) was usually taken as a geometrical probe to indicate enantioselectivity (Henke et al., 2003). The d_NE–O_ of Est924 with (*R*) and (*S*)-naproxen ethyl ester were measured in the most favorable binding mode. The Δd_NE–O_ of (*R*)-naproxen ethyl ester (3.3Å) was 0.3Å shorter than (*S*)-naproxen ethyl ester (3.6Å), which corresponded to the slight (*R*)-stereopreference of Est924. We superimposed the modeled structure of M3 and Est924, and found that the α-methyl of (*R*)-naproxen ethyl ester was blocked by the steric hindrance of residue W203, which prevented it from being effectively bound. In addition, the molecular docking results also suggested that (*S*)-naproxen ethyl ester was not affected by the newly generated steric hindrance from large side chain amino acids in M3. However, mutations reduced the activity pockets of enzymes which were not conducive to the entry of substrates, so their activities on (*S*)-naproxen ethyl ester were reduced. This suggested that the enantioselectivity reversal of the enzyme was due to the steric hindrance effect that made it difficult for the enzyme to bind (*R*) -enantiomer, which was consistent with the results of specific activity tests.

**TABLE 3:** Comparison of enzyme activity and enantioselectivity of Est924 and its mutants.

Entry	Esterase	Specific activity (mM/mg/min)^[a]^		Conversion/%^[b]^	*E* value^[c]^
		(*R*)-Naproxen ethyl ester	(*S*)-Naproxen ethyl ester		
1	Est924	3.5	3.3	45	1.3 (*R*)
2	A203W	0.5	3.1	38	7 (*S*)
3	G208F	0.8	3.2	33	4.6 (*S*)
4	M2	0.04	2.6	31	59 (*S*)
5	M3	0.02	2.5	30	68 (*S*)

*^[a]^Reactions were carried out under the optimal conditions.^[b]^Using racemic naproxen ethyl ester as substrate.^[c]^*E* values were calculated refers to the method of Ngo et al. (2019).*

### Resolution of Racemic Ibuprofen Ethyl Ester and Ketoprofen Ethyl Ester With M3

In addition to (*S*)-naproxen, (*S*)-ibuprofen and (*S*)-ketoprofen are also widely used. A variety of esterases have been used to produce them through the resolution of corresponding racemic esters (Kim et al., 2002; Choi et al., 2003; Sathishkumar et al., 2010; López et al., 2014; Yoon et al., 2014; Memarpoor-Yazdi et al., 2018). Est924 could effectively hydrolyze both racemic ibuprofen ethyl ester and ketoprofen ethyl ester (Table 1). The optimal variant (M3) was tested for enantioselective resolution of these racemic esters. The results showed that M3 preferred to hydrolyze their (*S*)-conformation esters, generating (*S*)-ibuprofen (74.5%*ee*_s_) and (*S*)-ketoprofen (95%*ee*_s_), respectively. These results showed that we successfully generated Est924 variants with inverted and highly increased enantioselectivity toward ethyl (*S*)-2-arylpropionates and provided Est924 mutants with potential application in industrial biocatalysts. On the other hand, the low enantioselectivity of M3 to ibuprofen ethyl ester also suggested that the stereoselectivity of the enzyme to ethyl 2-arylpropionates was also regulated by other residues in the active pocket, especially the acyl binding site.

### Synthesis of (*S*)-Ketoprofen and (*S*)-Naproxen by Whole-Cell Catalysis

**FIGURE 5:**
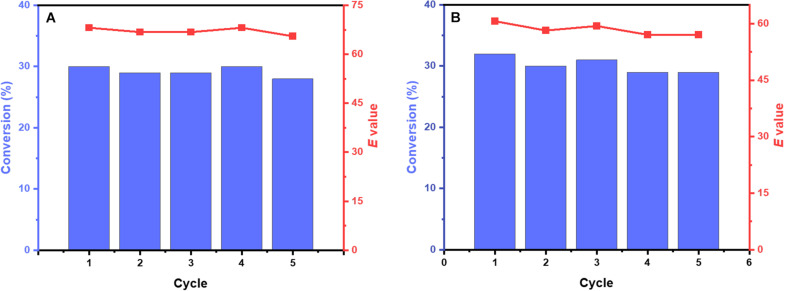
Repeated use of cells. The reaction mixture containing 50 mM potassium phosphate buffer (pH 8.0), 10 mM naproxen ethyl ester **(A)** or ketoprofen ethyl ester **(B)**, and 5% DMSO to form cell suspension (OD600 nm = 3) with a total volume of 10 mL (in a 50 mL shake flask). The bioconversion reactions were performed at 30°C and 200 rpm for 1 h (ketoprofen ethyl ester) and 1.5 h (naproxen ethyl ester) in each cycle respectively. Royal blue bars, conversion of *rac*-ketoprofen ethyl ester and naproxen ethyl ester; Salmon point-plot, *E* values of (*S*)-ketoprofen and (*S*)-naproxen.

Compared with free enzyme and immobilized enzyme, whole-cell catalysts avoided the separation and purification of enzymes, which might be the better method for reducing the cost of the process. Ketoprofen ethyl ester and naproxen ethyl ester were selected as substrates due to the high enantioselectivity of M3. Control cells harboring the empty vector (in the absence of M3) were verified to catalyze the conversion of ketoprofen ethyl ester and naproxen ethyl ester was less than 1% within 24 h, which indicated that the whole-cell catalyst did not have undesired reactions. Besides, although the cell envelope of the whole-cell catalysts helps stabilize enzymes, it reduces the mass transfer efficiency of substrates and products. Considering the activity and recovery of the whole-cell catalyst, freeze-thaw treatment of *E. coli* cells was a common method (Schwaiger et al., 2021). Using cell lysate as a reference, the cells achieved about 81% of the relative activity and still maintained high (*S*)-enantioselectivity toward ketoprofen ethyl ester (95%*ee*_S_) and naproxen ethyl ester (96%*ee*_S_) after a single freeze-thaw cycle. The recyclability of biocatalyst was an important factor in the industrial application of the whole-cell biocatalytic process. As shown in Figure 5, the cells could be reused for at least five cycles without sacrificing the activity and enantioselectivity.

## Conclusion

In this study, we carefully analyzed the structure of 41 wild-type esterases of the bHSL family. Their crystal structures are highly similar, although with only low to moderate sequence similarity. Several residues that may reverse and enhance the enantioselectivity of the enzyme toward (*S*)-2-aryl propionate esters were found through docking (*R*) and (*S*)-naproxen ethyl ester into the crystal structure of Est25. Est924, an esterase of the bHSL family, was set as the starting enzyme, because it could hydrolyze a variety of ethyl 2-arylpropionates with light (*R*)-enantioselectivity. By protein engineering, the key position A2 was substituted by tryptophan, and the substrate binding pockets were reshaped due to its large and hydrophobic side chain; this switched its stereoselectivity to (*S*)-2-aryl propionate esters. Another key residue at position B1 was mutated to phenylalanine, which further improved the stereoselectivity of the enzyme. Mutant M3 has A2 and B1 di-substitution, could resolve racemic naproxen ethyl ester, ketoprofen ethyl ester and ibuprofen ethyl ester with moderate to high (*S*)-enantioselectivity. In addition, an easy-to-perform whole-cell catalytic approach was successfully developed and used in the synthesis of (*S*)-ketoprofen and (*S*)-naproxen.

## Data Availability Statement

The datasets presented in this study can be found in online repositories. The names of the repository/repositories and accession number(s) can be found in the article/ Supplementary Material.

## Author Contributions

XL analyzed the structures, screened the key residues, and performed HPLC analysis. MZ constructed site mutations, screened clones, and tested activity and selectivity. XF written and revised the manuscript. YF conceived the study and supervised the experiments. All authors have read and approved the manuscript.

## Conflict of Interest

The authors declare that the research was conducted in the absence of any commercial or financial relationships that could be construed as a potential conflict of interest.

## Footnotes

1 https://swissmodel.expasy.org

2 http://services.mbi.ucla.edu/PROCHECK

## Abbreviations

*ee*: enantiomeric excess
IPTG: isopropyl- β-d -thiogalactopyranoside
NSAIDs: non-steroidal anti-inflammatory drugs.
