# Supplementary material for: Enhanced Production of (S)-2-arylpropionic Acids by Protein Engineering and Whole-Cell Catalysis
Source: Front Bioeng Biotechnol. 2021 Jul 7;9:697677. doi: 10.3389/fbioe.2021.697677 (PMC8293918; doi:10.3389/fbioe.2021.697677)

**Supporting Information**

**Enhanced production of  (S)-2-arylpropionic acids by protein engineering and whole-cell catalysis**

Xiaolong Liu^1&^, Meng Zhao^2&^, Xinjiong Fan^2^* and Yao Fu^1^*

^1^Hefei National Laboratory for Physical Sciences at the Microscale, CAS Key Laboratory of Urban Pollutant Conversion, Anhui Province Key Laboratory of Biomass Clean Energy, iChEM, University of Science and Technology of China, Hefei, Anhui 230026, China.

^2^School of Basic Medical Sciences, Anhui Medical University, 81 Meishan Rd., Hefei, 230032, Anhui, People’s Republic of China

^&^These authors contributed equally to this work.

*Correspondence should be addressed to:

Dr. Xinjiong Fan: fanxinjiong@126.com

Pro. Yao Fu: [fuyao@ustc.edu.cn](mailto:fuyao@ustc.edu.cn)

Number of pages: 10

Number of figures: 2

Number of tables: 3

**Table of Contents**

## General Information S3

1. **Supplementary Figures and Tables S4-S6**
2. **HPLC Data S7-S8**
3. **NMR Spectra S9-S11**
4. **General information**

Unless otherwise noted, reagents and organic solvents were obtained from chemical suppliers in reagent grade quality and used without further purification. All reactions were monitored by thin-layer chromatography (TLC) with Haiyang GF254 silica gel plates. Flash column chromatography was carried out using 100-200 mesh silica gel at increased pressure. For visualization, TLC plates were either placed under ultraviolet light, or stained with iodine vapor, or acidic vanillin. NMR spectra were recorded on a 400 MHz spectrometer.

## Supplementary Figures and Tables

**Table S1.** Sequences of mutagenesis primers used for construction of Est924 variants.

| Mutation | Forward primer^a^ | Reverse primer |
| --- | --- | --- |
| A203W | GACCCGATCATC**TGG**GGCGACCCGCAAGGCATTC | GAATGCCTTGCGGGTCGCC**CCA**GATGATCGGGTC |
| G208F | GACCCGCAA**TTT**ATTCGCGCGATCGG | CCGATCGCGCGAAT**AAA**TTGCGGGTC |
| G208H | GACCCGCAA**CAC**ATTCGCGCGATCGG | CCGATCGCGCGAAT**GTG**TTGCGGGTC |
| I212F | CATTCGCGCG**TTT**GGCGCCGTCTACAC | GTGTAGACGGCGCC**AAA**CGCGCGAATG |
| I202F | GACCCGATC**TTTTGG**GGCGACCCGC | GCGGGTCGCC**CCAAAA**GATCGGGTC |

^a^Bold letters indicate nucleotides that were exchanged.

Table S2. Chiral HPLC methods and retention times for ethyl 2-arylpropionates.

| Esters | Method | Rt_(_*_S_*_)_ min | Rt_(_*_R_*_)_ min |
| --- | --- | --- | --- |
|  | Chiralcel OD-H, hexane/2-propanol (98 : 2) flowing at 0.7 mL min^-1^, UV 214 nm | 10.534 | 9.465 |
|  | Chiralcel OJ-H, hexane/2-propanol (99 : 1) flowing at 0.7 mL min^-1^, UV 214 nm | 8.312 | 10.330 |
|  | Chiralcel OJ-H, hexane/2-propanol (99 : 1) flowing at 0.7 mL min^-1^, UV 214 nm | 27.046 | 28.804 |

**
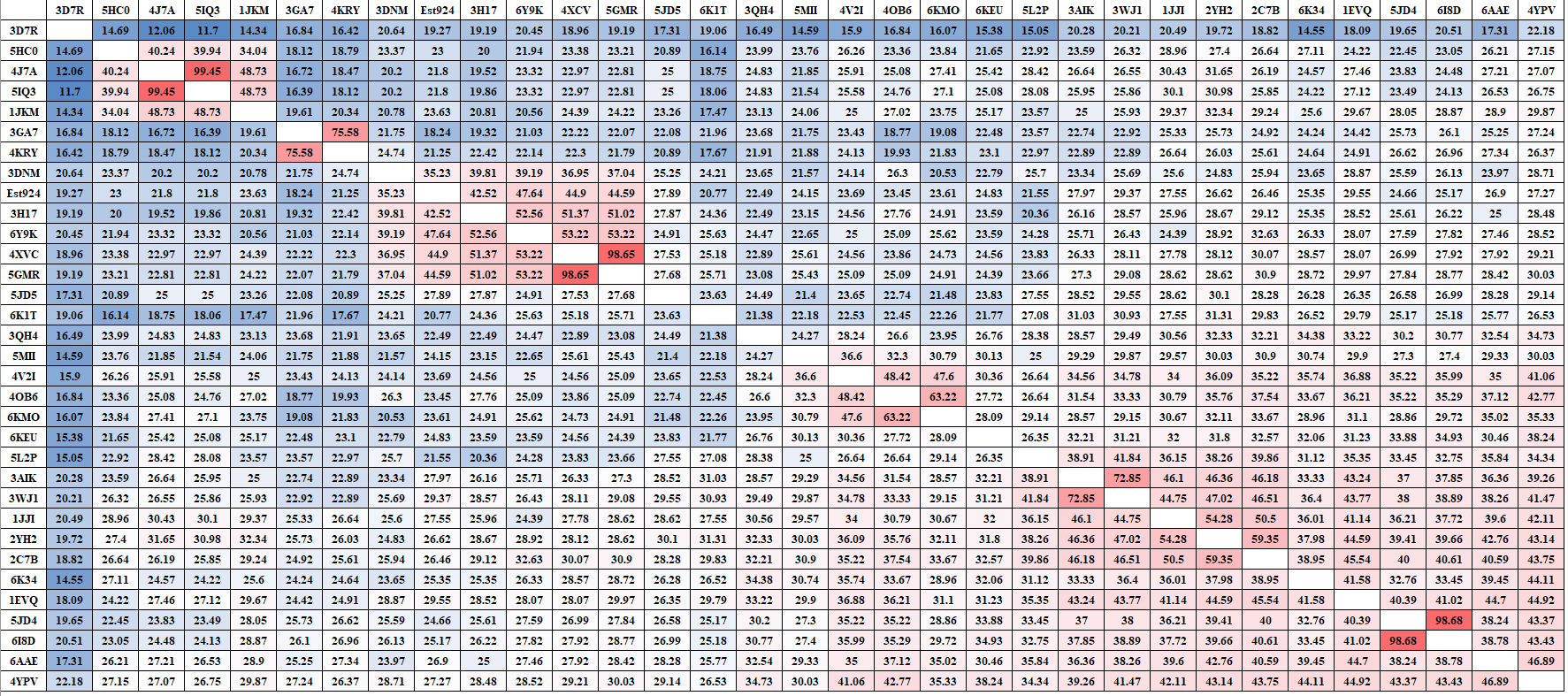
Table S3.** The pairwise sequence identity analysis of full length esterases of bHSL family.

^a^Multiple sequence alignment and percent sequence identity matrix was created by Clustal Omega.

**
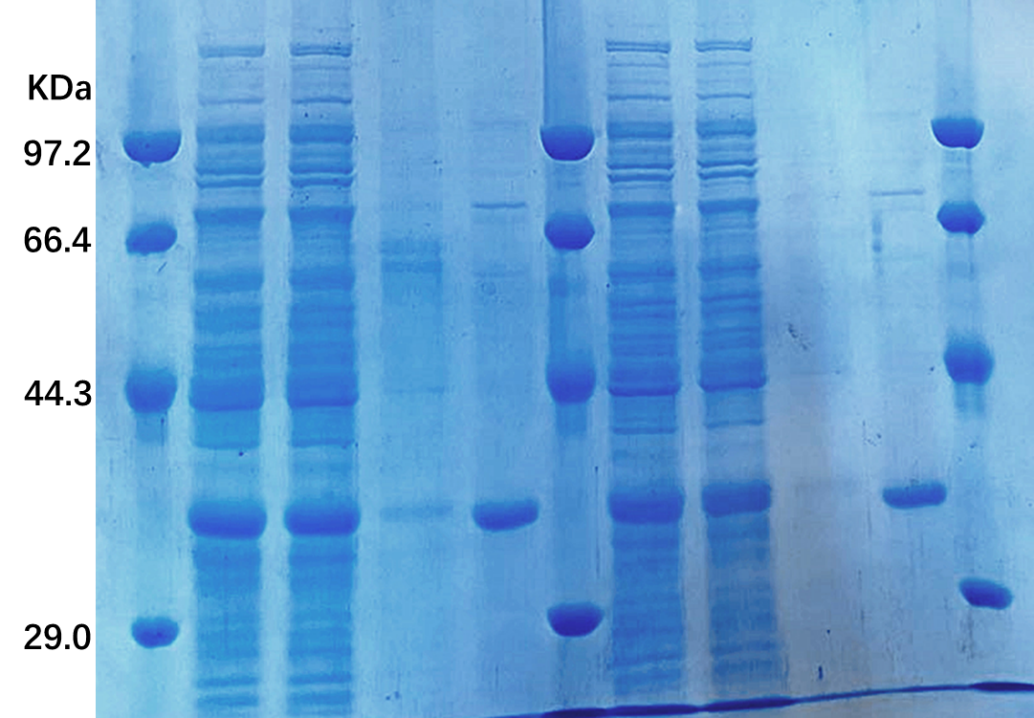
**

**Supplementary Figure S1.** SDS-PAGE analysis of the purified recombinant Est924 and M3 (I202F/A203W/G208F). Protein markers (lane 1, 6 and 11) stained with Coomassie blue, cell-free lysates (lane 2 and 7), the supernatant of *E. coli* BL21 (DE3) cell lysates (lane 3 and 8), the precipitate of the cell-free lysates (lane 3 and 9), purified target protein (lane 5 and 10). The molecular mass of the enzyme subunit was estimated using the following protein markers as standards: rabbit muscle phosphorylase B (97,200 Da); bovine serum albumin (66,409 Da); ovalbumin (44,287 Da); carbonic anhydrase (29,000 Da).


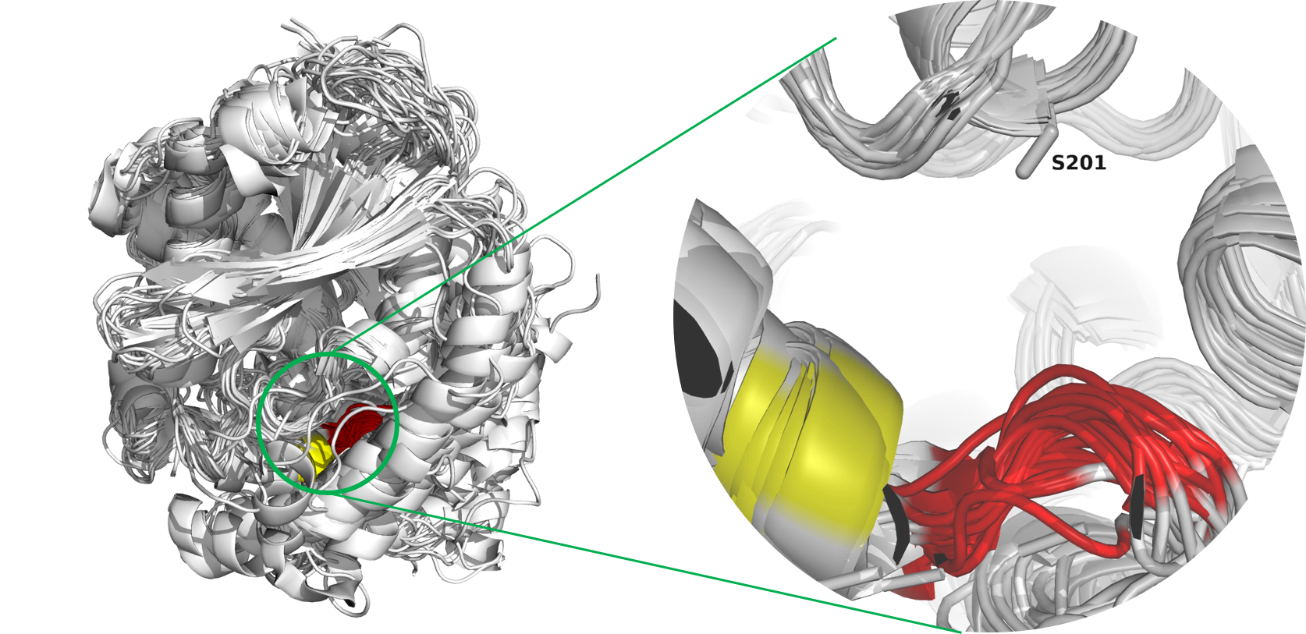


**Supplementary Figure S2.** Superimposed of the crystal structures of 33 bHSL family esterases. The positions of key residues A2 and B1 are shown in red and yellow, respectively.

**3. HPLC data**


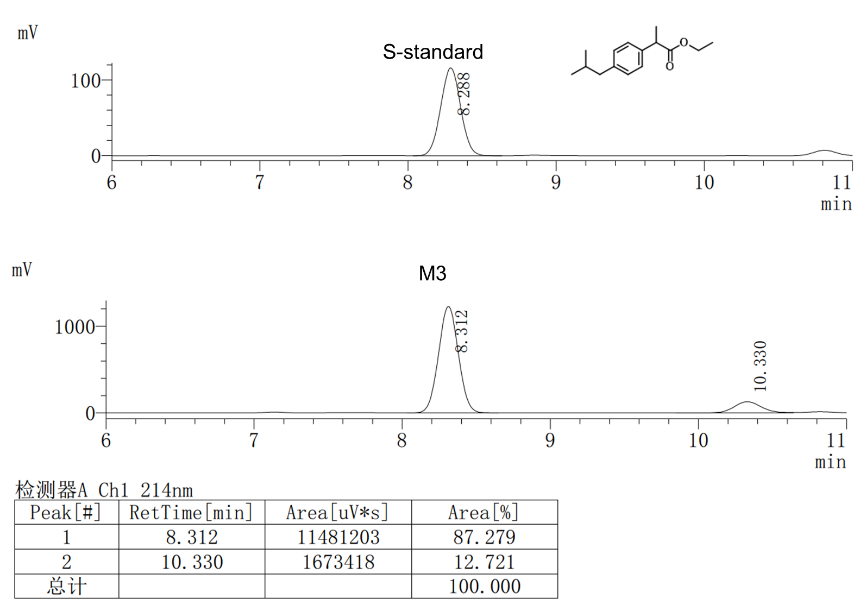


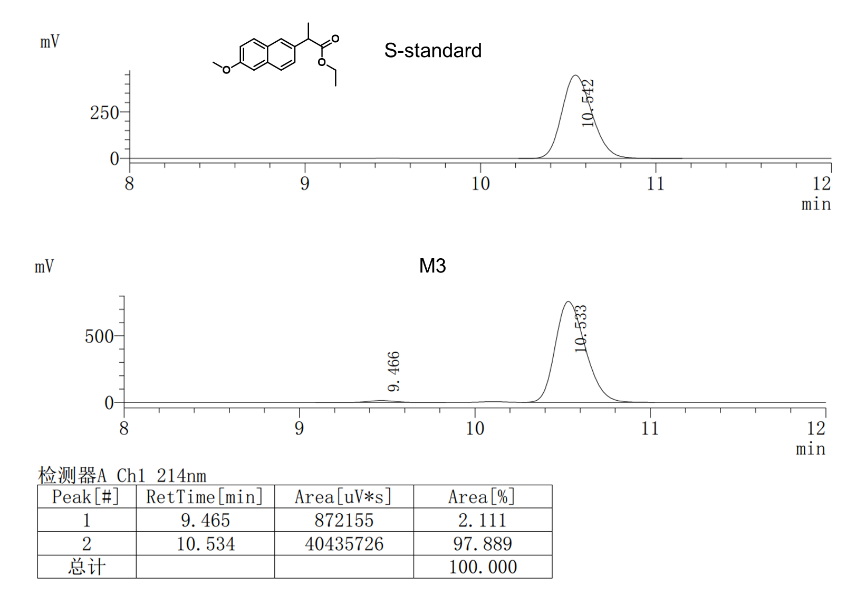


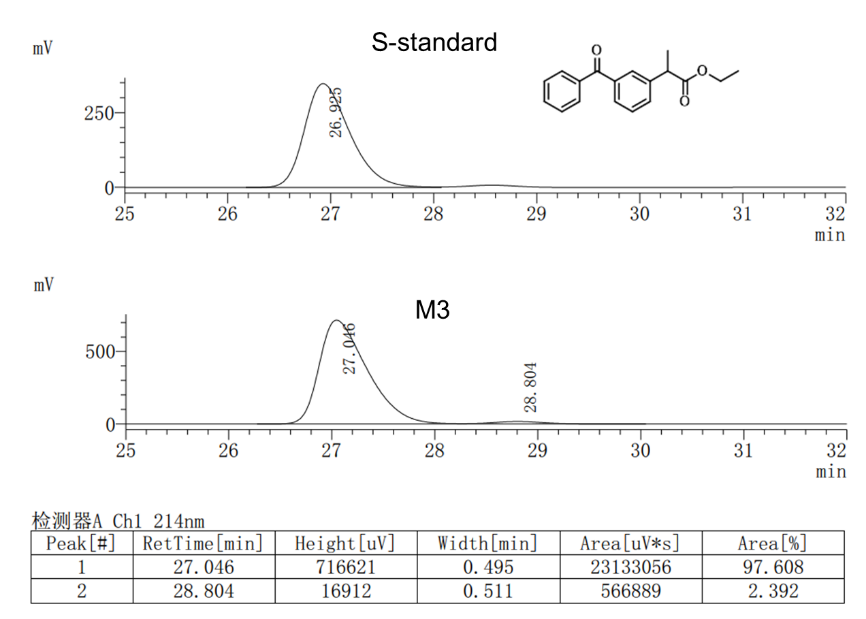


**4. NMR Spectra**

^1^H NMR (400 MHz, CDCl_3_) δ 7.20 (d, *J* = 8.1 Hz, 2H), 7.09 (d, *J* = 8.1 Hz, 2H), 4.12 (qq, *J* = 10.8, 7.1 Hz, 2H), 3.67 (q, *J* = 7.2 Hz, 1H), 2.44 (d, *J* = 7.2 Hz, 2H), 1.84 (dp, *J* = 13.7, 6.7 Hz, 1H), 1.48 (d, *J* = 7.2 Hz, 3H), 1.21 (t, *J* = 7.1 Hz, 3H), 0.89 (d, *J* = 6.6 Hz, 6H).

^13^C NMR (101 MHz, CDCl_3_) δ 174.77, 140.43, 137.91, 129.28, 127.12, 60.62, 45.18, 45.05, 30.16, 22.38, 18.59, 14.12.


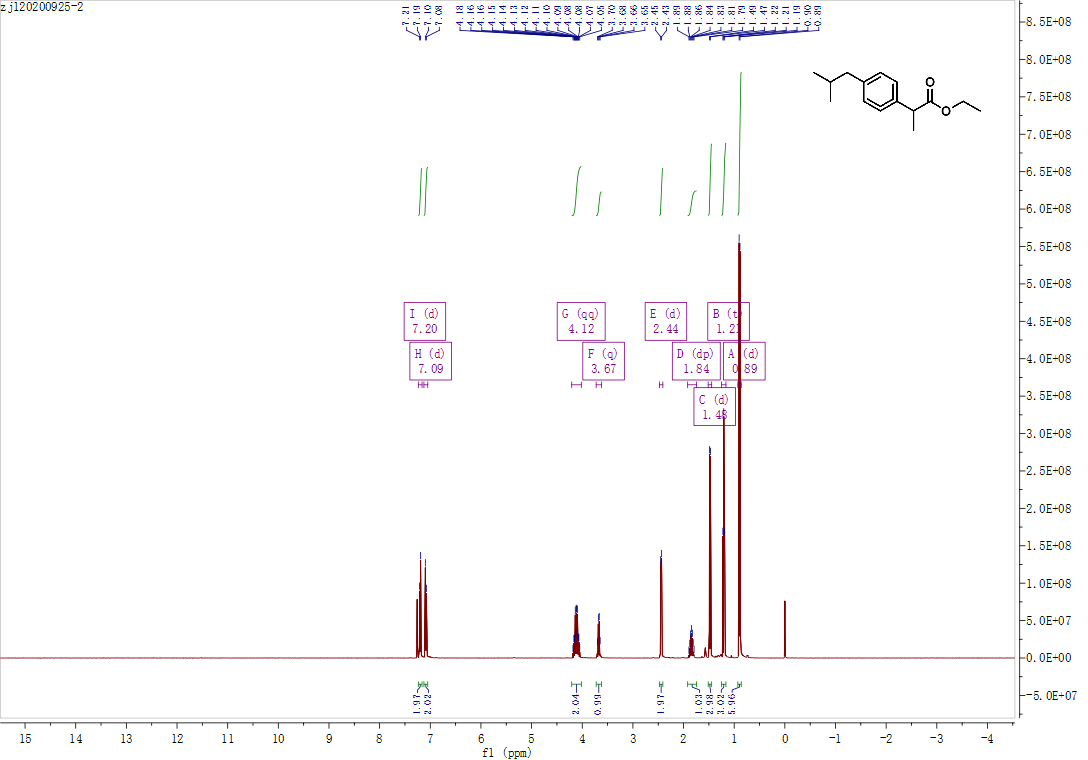


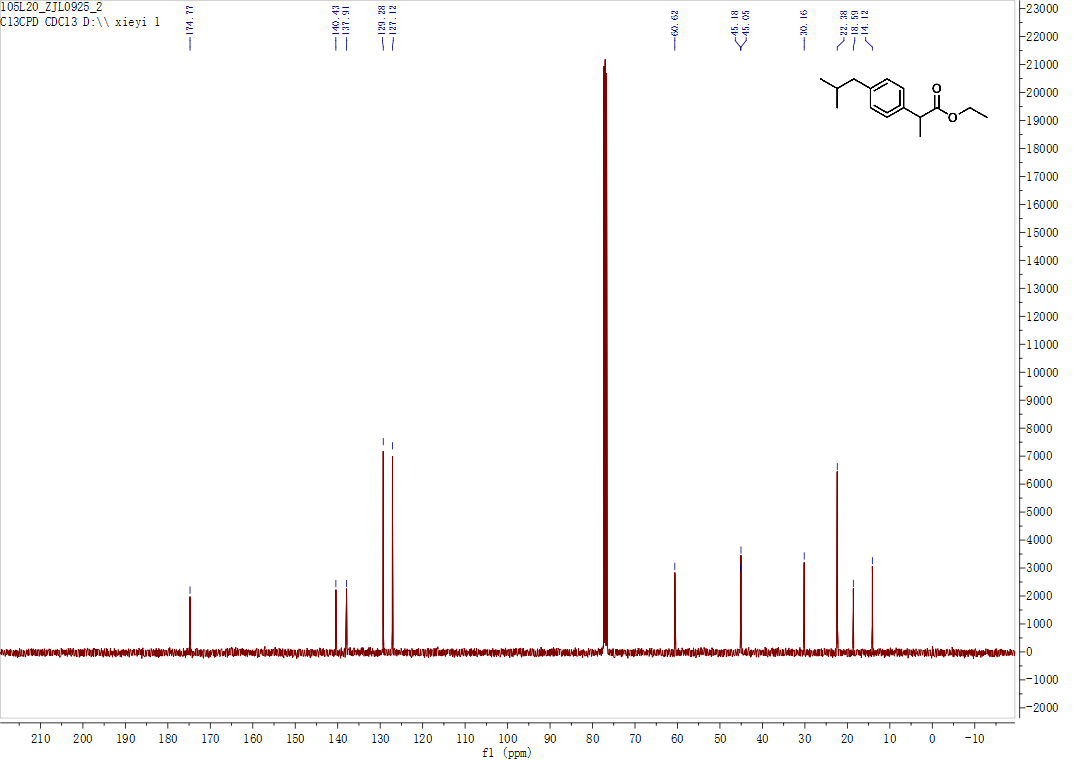

^1^H NMR (400 MHz, CDCl_3_) δ 7.84 – 7.65 (m, 4H), 7.64 – 7.52 (m, 2H), 7.52 – 7.40 (m, 3H), 4.21 – 4.06 (m, 2H), 3.79 (q, *J* = 7.2 Hz, 1H), 1.53 (d, *J* = 7.2 Hz, 3H), 1.22 (t, *J* = 7.1 Hz, 3H).

^13^C NMR (101 MHz, CDCl_3_) δ 196.51, 174.06, 140.98, 137.90, 137.56, 132.47, 131.49, 130.06, 129.22, 128.93, 128.52, 128.29, 60.93, 45.44, 18.51, 14.12.


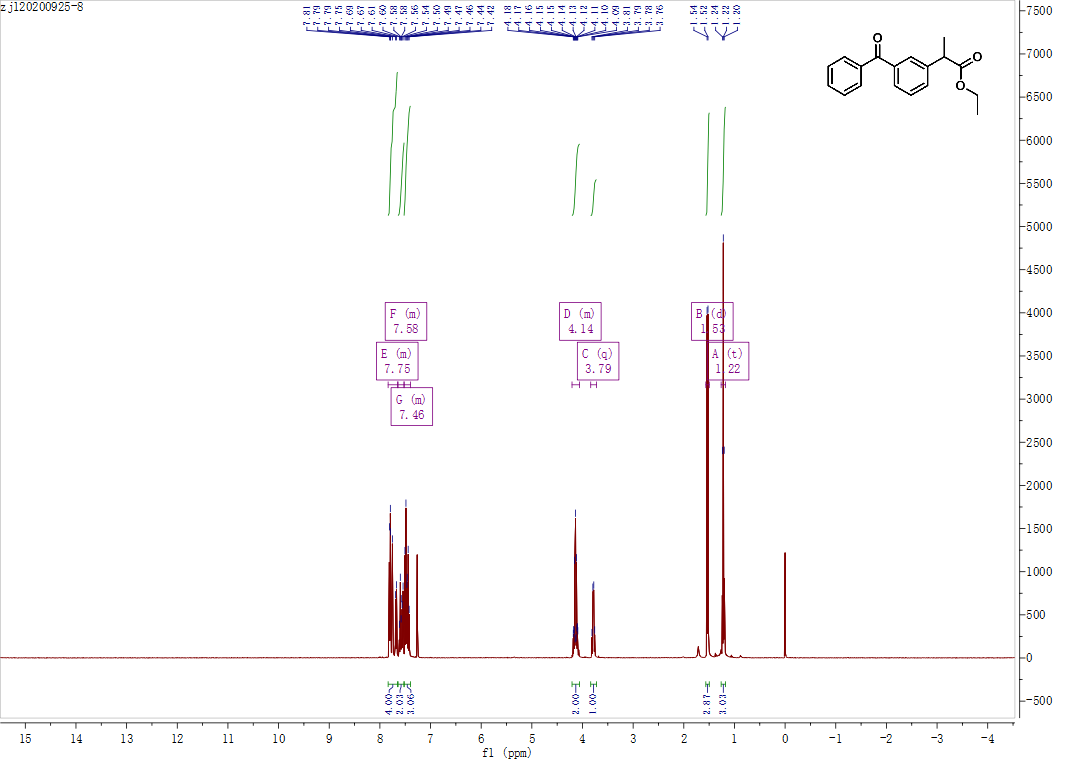


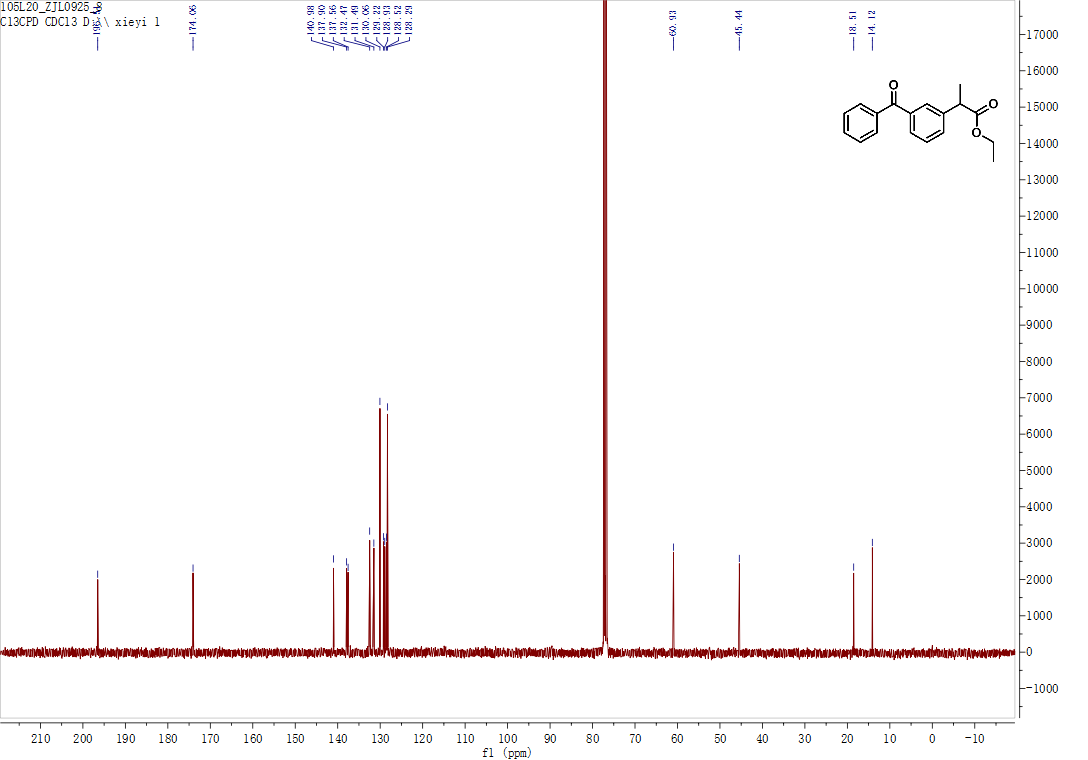

^1^H NMR (400 MHz, CDCl_3_) δ 7.73 – 7.62 (m, 3H), 7.41 (dd, *J* = 8.5, 1.7 Hz, 1H), 7.17 – 7.06 (m, 2H), 4.12 (qq, *J* = 10.8, 7.1 Hz, 2H), 3.92 – 3.74 (m, 4H), 1.57 (d, *J* = 7.2 Hz, 3H), 1.19 (t, *J* = 7.1 Hz, 3H).

^13^C NMR (101 MHz, CDCl_3_) δ 174.70, 157.65, 135.88, 133.69, 129.29, 128.97, 127.11, 126.26, 125.92, 118.94, 105.64, 60.75, 55.30, 45.51, 18.62, 14.15.


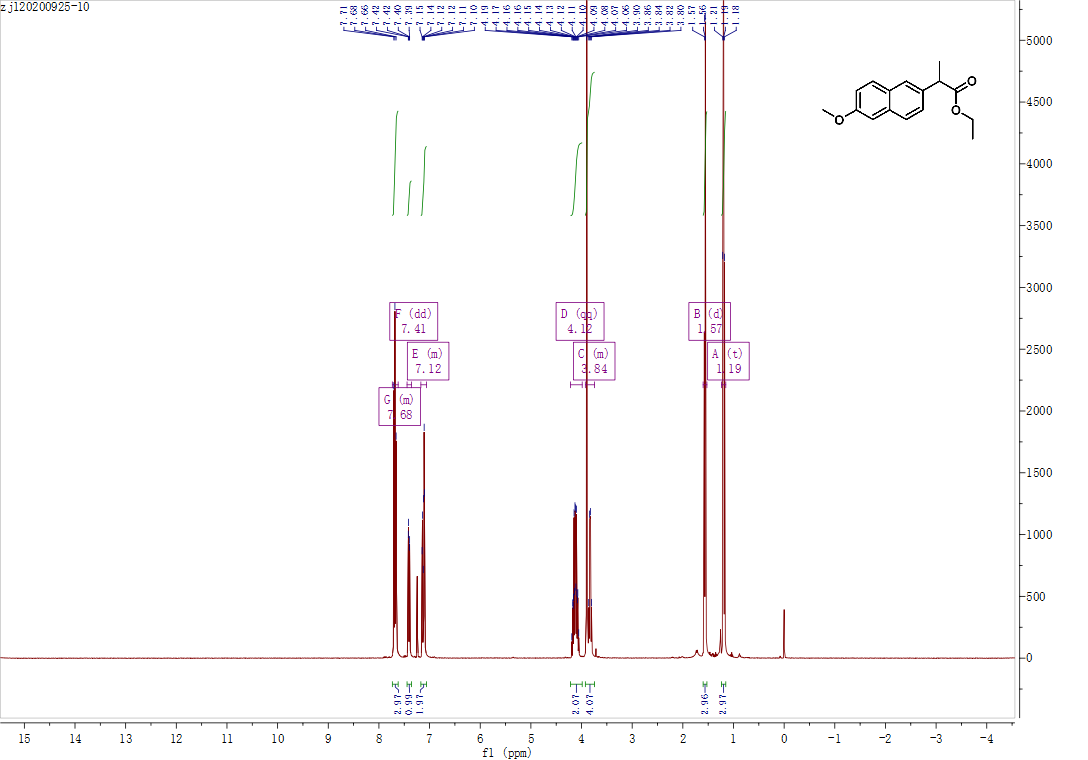


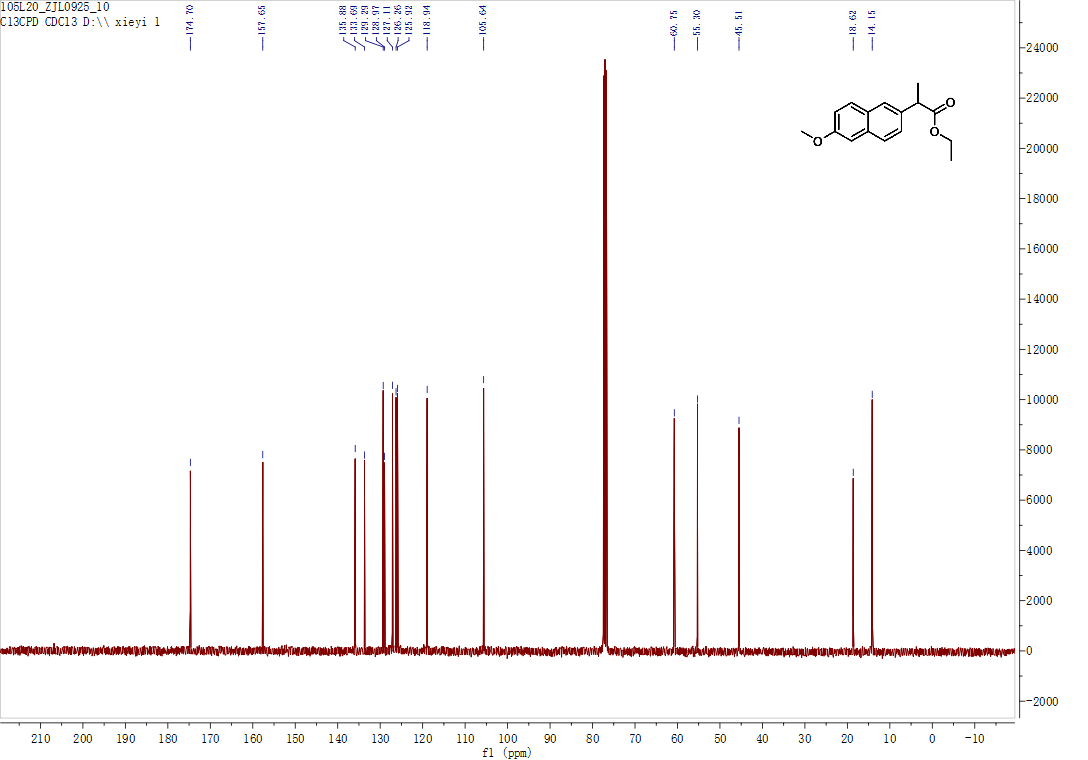

Supplement: Supplementary file 1 [file Table_1.DOCX]
